# Nondestructive Testing of Egg Freshness Based on Laser Doppler Vibrometry

**DOI:** 10.3390/s26175389

**Published:** 2026-08-26

**Authors:** Jingwei Zhang, Fulong Dong, Chan Wang, Wen Sun, Xiaojie Zhou

**Affiliations:** 1 School of Electrical and Electronic Engineering, Anhui Science and Technology University, Chuzhou 239000, China; wangc@ahstu.edu.cn (C.W.); sunw@ahstu.edu.cn (W.S.); zhouxiaoj@ahstu.edu.cn (X.Z.); 2 College of Intelligent Manufacturing, Anhui Science and Technology University, Chuzhou 239000, China; alone79@163.com

**Keywords:** egg freshness, laser Doppler vibrometry, nondestructive testing, machine learning

## Abstract

This study proposes a nondestructive method for evaluating egg freshness using laser Doppler vibrometry. A vibration measurement platform was constructed to collect vibration signals from intact eggs representing four freshness grades. The vibration signals were processed using multiplicative scatter correction, standard normal variate transformation, moving average filtering, Savitzky–Golay smoothing, and first-order Savitzky–Golay differentiation. Principal component analysis, successive projections algorithm, and competitive adaptive reweighted sampling were then used for feature extraction and dimensionality reduction. Six classification models were developed and compared, including support vector machine, k-nearest neighbor, random forest, naive Bayes, discriminant analysis, and linear discriminant analysis. The model based on moving average filtering, successive projections algorithm, and discriminant analysis using measurements from three egg locations achieved a classification accuracy of 96.7%. The model based on moving average filtering, competitive adaptive reweighted sampling, and random forest using blunt-end measurements also achieved an accuracy of 96.7%. These results demonstrate that laser Doppler vibrometry combined with machine learning can distinguish eggs with different freshness levels, providing a promising nondestructive approach for egg-quality evaluation.

## 1. Introduction

Eggs are rich in protein, fat, minerals, a variety of vitamins, trace elements, and a variety of essential amino acids, and are an indispensable treasure trove of human nutrition [1]. Eggs are often stored, transported, processed, and sold in the form of shelled eggs. However, as storage time increases and storage conditions change, the structure and internal quality of eggs deteriorate. Their shell and inner membrane gradually lose their protective effect, pathogenic microorganisms reproduce and grow, nutrients gradually decompose, and nutritional value is greatly reduced [2,3]. Furthermore, the deterioration of egg quality can affect consumers’ willingness to purchase, thereby causing significant economic losses to the agricultural and food industries. Therefore, to ensure the large-scale development of the egg industry, it is necessary to conduct a comprehensive assessment of egg freshness.

The freshness of eggs can be assessed using indicators such as Haugh unit (*HU*), air cell height, yolk coefficient, and albumen pH [4,5,6,7,8,9]. Although these methods provide reliable information about internal egg quality, they require breaking the eggs and are therefore unsuitable for rapid, large-scale inspection. To overcome this limitation, several nondestructive techniques have been developed, including manual light inspection, computer vision, electronic nose detection, and spectral analysis.

As eggs lose freshness, the yolk becomes disorganized, and the air cell expands; therefore, freshness can be assessed by visually examining the outlines of the yolk and air cell when the egg is held up to a strong light; however, this method is often influenced by the inspector’s experience. Machine vision can be integrated into automated sorting systems and has been used to classify eggs based on storage time and weight [10,11,12]. However, the quality of transmitted light images is easily affected by factors such as eggshell color, shell thickness, surface blemishes, lighting conditions, and limited visibility of the internal structure.

Electronic-nose systems are capable of detecting methyl mercaptan-type volatile compounds associated with egg spoilage [13,14,15], but their signals are susceptible to interference from ambient gases, humidity, temperature, sensor drift, and system contamination, which can reduce their robustness in real-world environments.

Spectroscopic techniques, including visible–near-infrared spectroscopy, hyperspectral imaging, and Raman spectroscopy, provide information related to the chemical and structural properties of eggs and have demonstrated promising classification or prediction performance [16,17,18]. However, these methods generally require strict control of illumination, optical geometry, spectral calibration, and measurement conditions.

In addition, techniques such as conductivity [19,20], dielectric spectroscopy [21], low-field nuclear magnetic resonance [22], and infrared thermography [23,24,25] can also provide useful information regarding egg freshness, but their applications may be limited by equipment cost, operational complexity, eggshell-related effects, or the need for specialized measurement environments.

In comparison with these methods, laser Doppler vibrometry (LDV) is a noncontact technique capable of measuring surface vibrations of objects with high sensitivity and temporal resolution, thereby enabling the assessment of their internal structure. Previous studies have demonstrated that LDV can be used to evaluate the texture, ripeness, and internal defects of agricultural crops such as apples, kiwifruit, and onions [26,27,28]. However, its application to the non-destructive assessment of egg freshness has not yet received sufficient attention. During storage, changes in egg white viscosity, yolk structure, and air cell characteristics may alter the dynamic response of the eggshell [29]. Therefore, this study aims to investigate whether LDV can effectively distinguish between eggs of different freshness grades.

## 2. Materials and Methods

### 2.1. Sample Preparation

The sample eggs were purchased from the Nanjing Panchu Mechanized Poultry Farm in Nanjing, Jiangsu Province, China. They were all from the same batch of visually intact, clean, and undamaged pink-shelled free-range eggs, which had been laid less than 24 h prior. Within 24 h of collection, visible dust and surface impurities were gently removed from the eggshells using a soft-bristled brush; the eggs were not washed with water, detergent, or any other liquid. The eggs were then visually inspected, and those with fine cracks were discarded. Ultimately, 600 eggs (39.05 ± 7.55 g) were selected for the experiment.

The eggs were divided into two groups: a vibration testing group consisting of 400 eggs, used to collect vibration characteristics of eggs with different freshness grades; and a calibration group consisting of 200 eggs, from which 5 eggs were randomly selected every 2 days to measure the *HU* [30]:

(1) $$HU=100\times \log_{10}(h-1.7w^{0.37}+7.57)$$

where *HU* is the Haugh unit, *h* is the height of the thick albumen (mm), and *w* is the weight of the egg (g).

According to internationally recognized egg freshness standards [31], the eggs were classified into four grades based on their *HU* values: AA grade (*HU* ≥ 72), indicating the highest freshness; A grade (60 ≤ *HU* < 72), generally considered suitable for routine culinary use; B grade (30 ≤ *HU* < 60), characterized by relatively weak and watery albumen and mainly used for further processing; and C grade (*HU* < 30), indicating poor freshness or staleness.

During storage, when eggs were classified as B-grade in terms of freshness, they were placed in an environment with a temperature of 25 °C and 100% humidity immediately after testing to accelerate the spoilage process and produce eggs with C-grade freshness.

### 2.2. LDV Vibration Detection System

**Figure 1:**
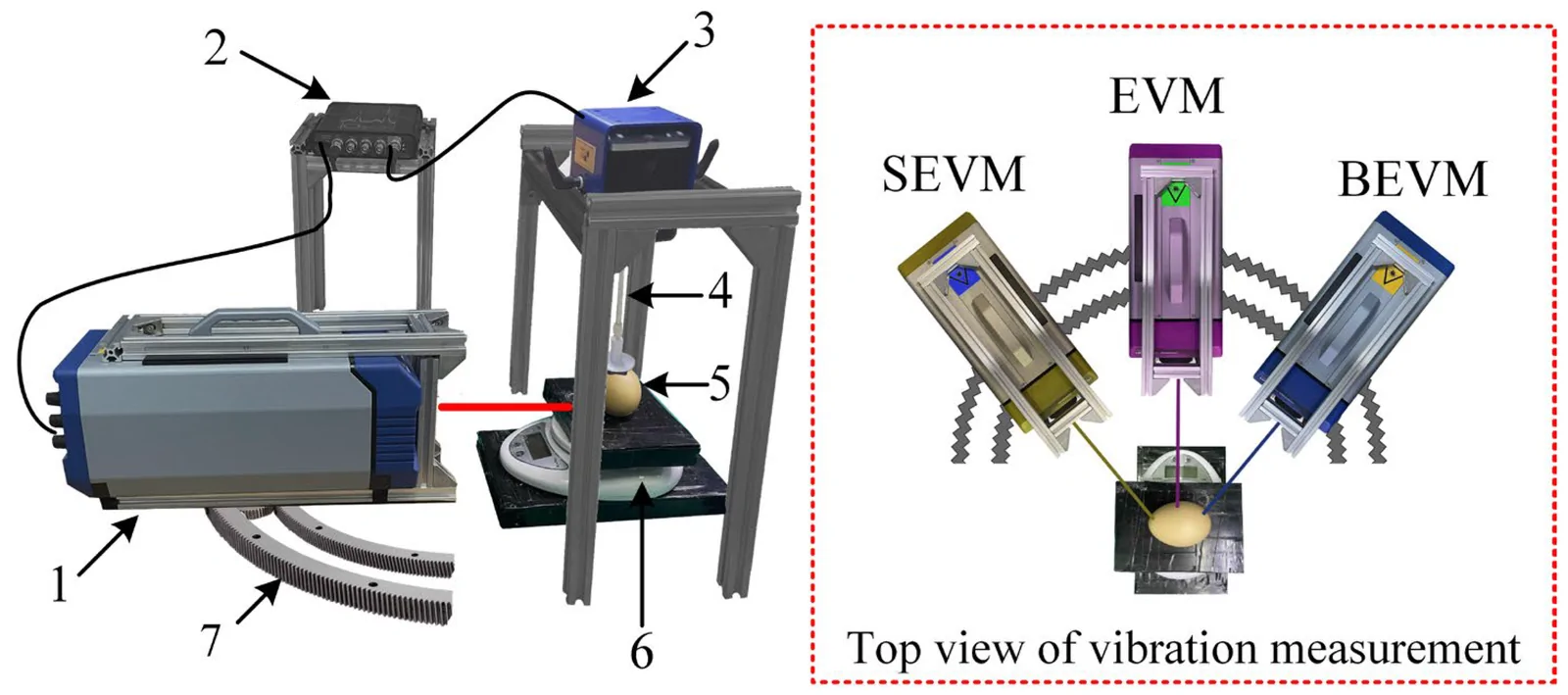
Egg vibration characteristics detection platform: (**1**) laser Doppler vibrometer; (**2**) dynamic signal analyzer; (**3**) exciter; (**4**) vibration-conducting hardware; (**5**) pink-shell egg; (**6**) pressure measuring device; (**7**) circular track; (**SEVM**) sharp-end vibration measurement; (**EVM**) equatorial vibration measurement; (**BEVM**) blunt-end vibration measurement.

A laser Doppler vibration measurement system was used to collect vibration data from eggs (Figure 1). The system consisted of a laser Doppler vibrometer (OptoMET Vector-Series, digital, Germany), a miniature electric shaker (PCB 2004E, New York, ny, USA), a dynamic signal analyzer (IOtech 640u, Beijing, China), a pressure-measuring device, and a computer for processing vibration signals (MATLAB R2018b).

The PCB 2004E applied a broadband sinusoidal linear sweep vibration at 8000 Hz to the equatorial region of the egg, with an excitation duration of 0.1 s, while maintaining the contact force at 5.0 ± 0.1 N using a force measurement device. With a velocity resolution of 10 nm/s (Table 1), OptoMETVector could reliably detect minute vibrations in the blunt end, sharp end, and equatorial measurement regions of an egg. The IOtech 640u was used to acquire the vibration characteristics and output them to the host computer for processing.

**Figure 2:**
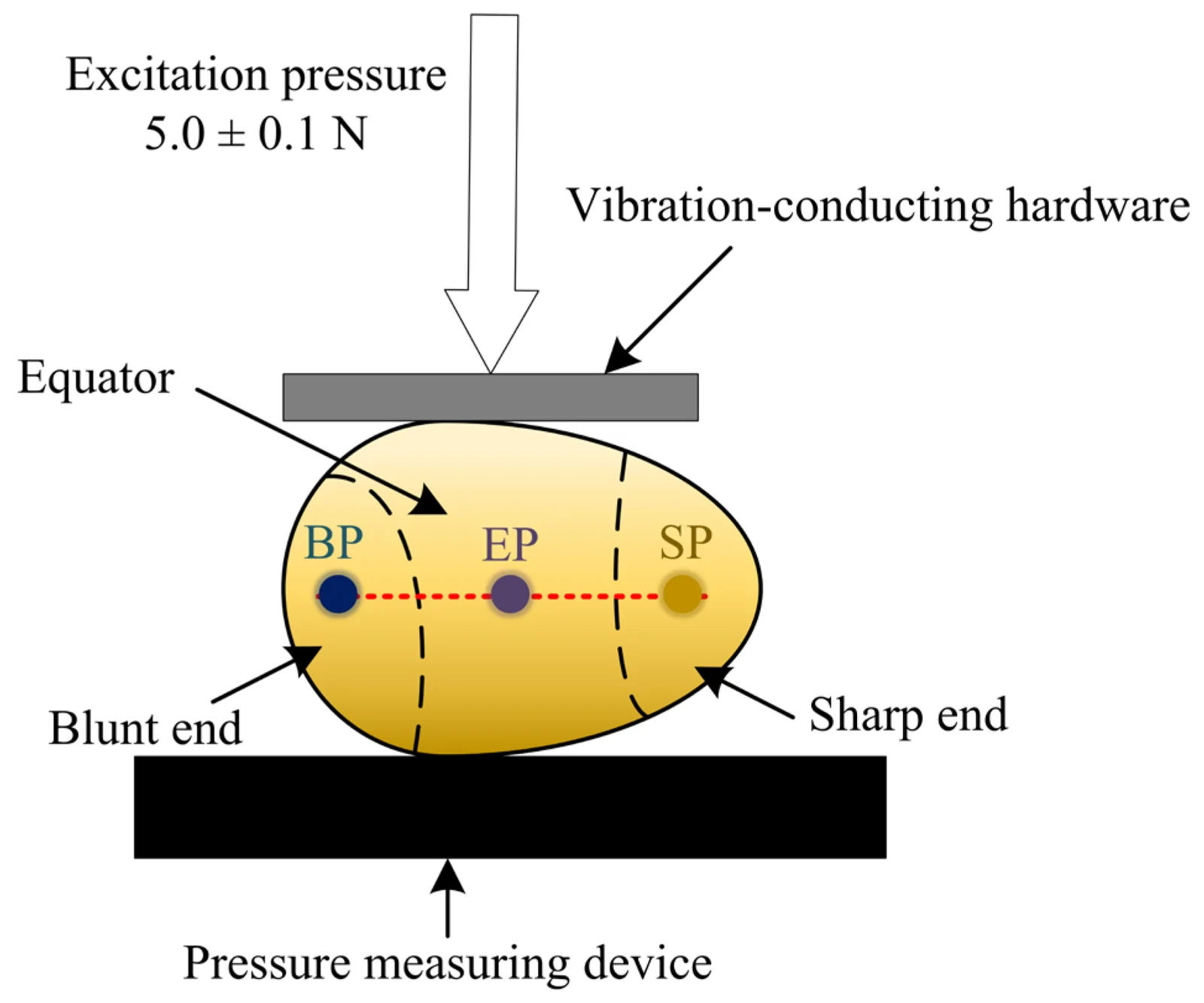
Schematic diagram of egg vibration testing: (**BP**) blunt-end measurement point; (**SP**) sharp-end measurement point; (**EP**) equatorial measurement points.

All experiments were conducted with the eggs placed horizontally, with the excitation pressure always perpendicular to the LDV measurement direction (Figure 2). The laser Doppler vibrometer moved laterally along a slide rail, directing the laser beam at the large end, small end, and equatorial regions of the egg to obtain vibration data. It should be noted that the vibration system must ensure that the excitation applied is exactly the same each time. Since the pressure measuring device is located beneath the egg, the initial pressure value corresponds to the egg’s weight; therefore, the pressure must be zeroed before testing different eggs. In addition, to obtain frequency response curves with good repeatability, we conduct multiple tests on the same vibration measurement area.

### 2.3. Egg Freshness and Vibration Signal Acquisition

**Figure 3:**
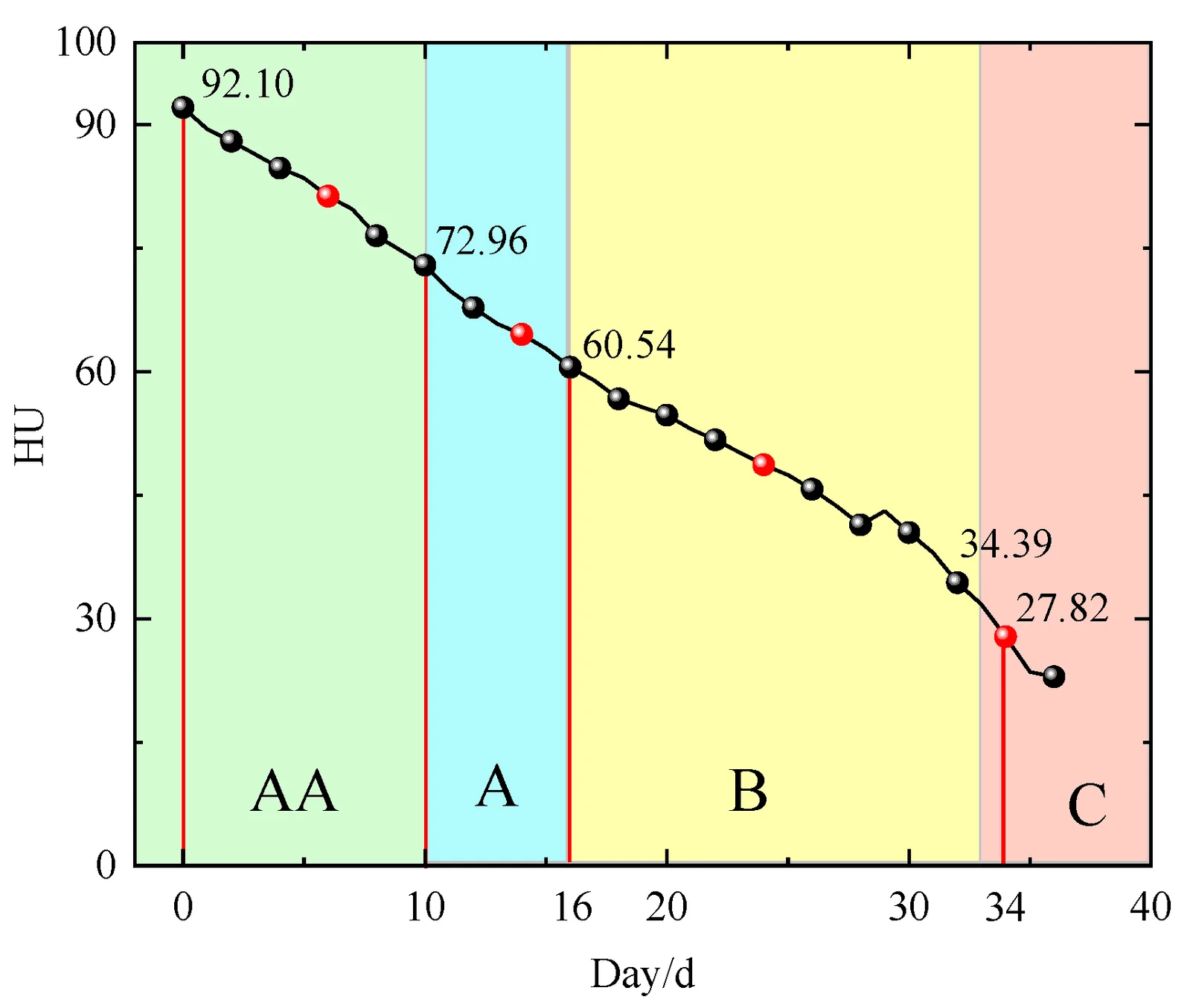
Egg Haugh unit curve.

To determine the freshness of the eggs, the calibration group randomly selected five eggs every two days to measure the *HU* and plotted a curve showing how this value changed over storage time (Figure 3). We randomly selected sets of eggs on Day 6 (AA), Day 14 (A), Day 24 (B), and Day 34 (C), and measured their vibration characteristics at the blunt end, sharp end, and equator of each egg.

### 2.4. Power Spectrum Density (PSD) of Vibration Signals

The vibration signals generated when eggs were excited could be regarded as random stationary signals, and their PSD is the fast Fourier transform (FFT) of the autocorrelation function of the random stationary signal [32]:

(2) $$S_{x}(\omega )=\int_{-\infty }^{\infty }R_{x}(\tau )e^{-j\omega \tau }d\tau$$

where *S_x_*(*ω*) is the power spectral density of the egg’s vibration signal *x*(*t*), *ω* is the angular frequency, *R_x_*(*τ*) is the autocorrelation function of *x*(*t*), and *τ* represents the time lag. The power spectral density was estimated numerically using the Welch method based on the measured LDV vibration signals.

**Figure 4:**
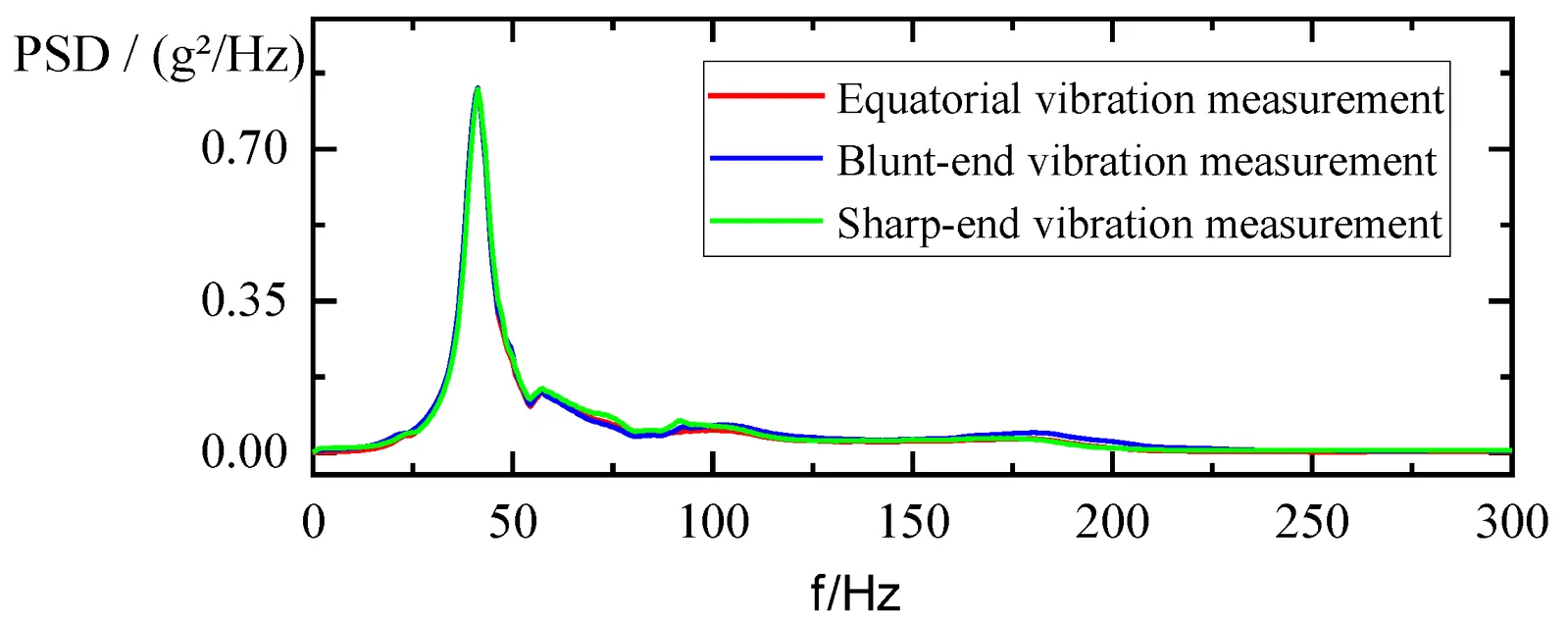
Power spectral density (PSD) curve (AA).

After estimating the PSD of the egg vibration data and normalizing the data, the average power spectrum for each measurement point was calculated, thereby obtaining the vibration information of the egg (Figure 4). Under a 5 N force-sweep vibration, the PSD curve obtained from single-point vibration measurements of AA-grade eggs exhibits relatively pronounced fluctuations primarily in the 0–250 Hz frequency band; therefore, eigenvalue extraction can be conducted primarily within this frequency band. For the three-point measurement condition, the PSD feature vectors obtained from the blunt end, sharp end, and equatorial region of the same egg were concatenated in a fixed order to form one combined feature vector.

### 2.5. Feature Processing and Egg Freshness Modeling

Since the PSD curve of egg vibrations may be affected by instrument noise, environmental vibrations, baseline shifts, and amplitude variations among individual eggs, five preprocessing methods are discussed [33,34,35,36,37]. Multiplicative scatter correction (MSC) and standard normal variate (SNV) were selected to reduce amplitude variations in the PSD curve caused by eggshell curvature. Moving average filtering (MAF) and Savitzky–Golay smoothing (SG) were chosen to suppress random noise while preserving the main spectral features. The Savitzky–Golay first derivative (SG-FD) was introduced to enhance local variations in the PSD curve and reduce the impact of baseline drift.

After preprocessing, three feature dimension-reduction methods with complementary strategies were employed. Principal component analysis (PCA) was used to compress the relevant variables and explore the primary structure of the vibration data, thereby addressing the issues of high-dimensional redundancy and noise interference in the egg vibration data [38]. The successive projection algorithm (SPA) was used to select a relatively small number of variables with low mutual correlation, facilitating the construction of a lightweight model with high interpretability [39]. Competitive adaptive reweighted sampling (CARS), as a supervised variable selection method, was used to retain variables that contribute significantly to the calibration model, thereby addressing the issues of key feature selection and overfitting [40].

Finally, we compared the performance of six models for detecting egg freshness: support vector machines (SVM) [41], discriminant analysis classifier (DAC) [42], latent Dirichlet allocation (LDA) [43], k-nearest neighbor (KNN) [44], random forest (RF) [45], and naive Bayes (NB) [46], thereby evaluating the contributions of preprocessing and feature selection methods within a consistent analytical framework.

To prevent information leakage, the samples were first divided into a training set and an independent test set. A total of 70% of the samples were assigned to the training set, and the remaining 30% were assigned to the independent test set. All preprocessing parameters were estimated using the training set only. PCA, SPA, and CARS feature extraction were also performed exclusively on the training set. The independent test set was used only once for the final evaluation of classification performance.

## 3. Results

### 3.1. PCA-Based Feature Extraction and Model Development

**Figure 5:**
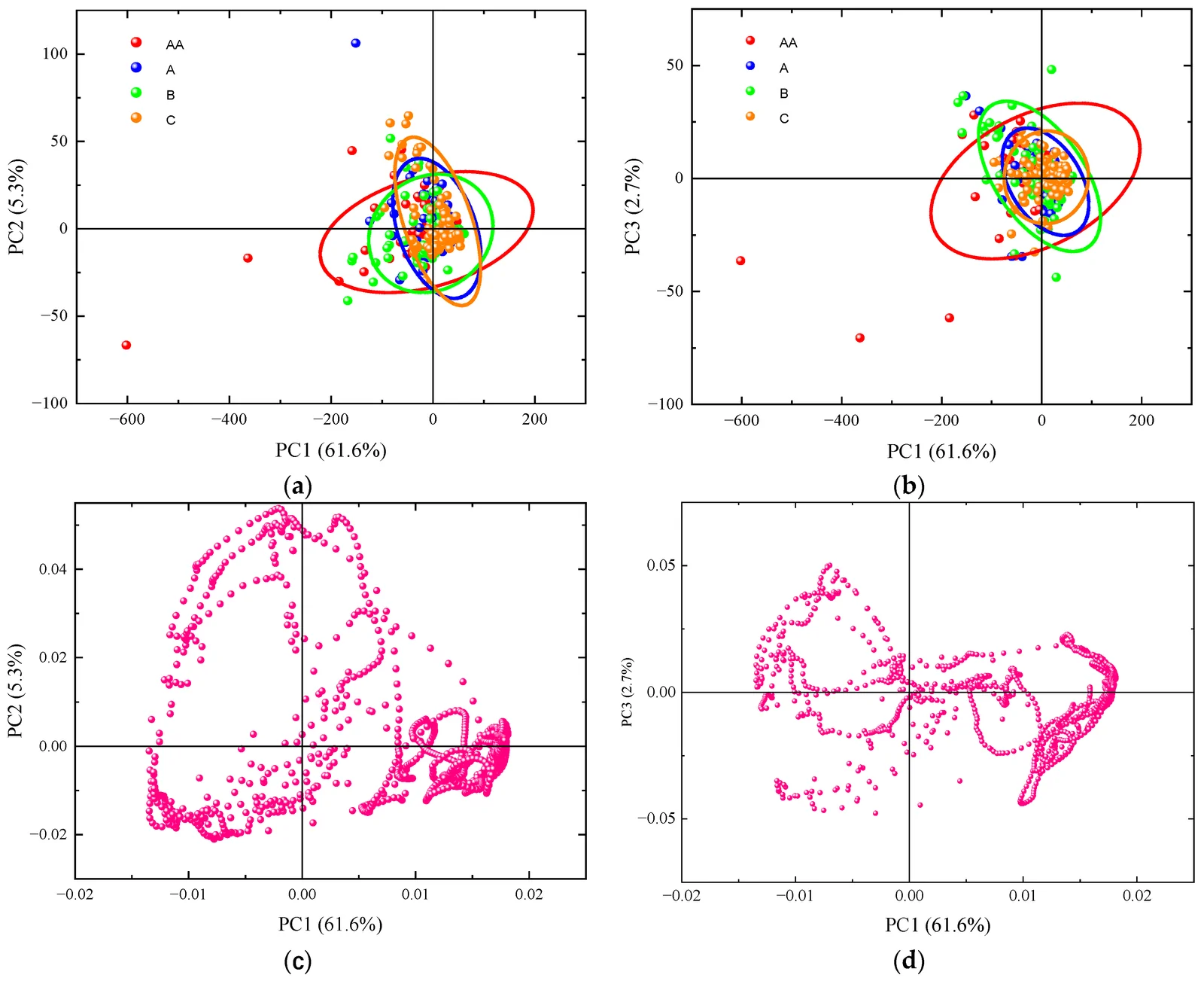
PCA of three-point egg vibrations based on SG-FD preprocessing: (**a**) score plot of PC1–PC2; (**b**) score plot of PC1–PC3; (**c**) loading plot of PC1–PC2; (**d**) loading plot of PC1–PC3.

PCA transforms the preprocessed, correlated variables into a set of mutually orthogonal principal components, enabling data compression and exploratory visualization. The score plots were used to examine the overall distribution of AA, A, B, and C, whereas the principal-component scores were also used as input features for subsequent classification models. Figure 5 shows the results of PCA (PC1–PC2, PC1–PC3) based on SG-FD preprocessing and three-point vibration measurement conditions, illustrating the distribution of eggs with different freshness grades and the degree of their separation. PC1, PC2, and PC3 denote the first, second, and third principal components, respectively. PC1 accounts for the largest proportion of the total variance, followed by PC2 and PC3.

Figure 5a,b show the score plots for the AA, A, B, and C grades of eggs, indicating that C-grade eggs are easily distinguishable from eggs in other freshness grades, while there is significant overlap between AA and A. In other words, while we can use PCA to distinguish edible eggs from spoiled eggs, it is difficult to distinguish between more specific freshness grades; therefore, the accuracy of PCA may not be suitable for subsequent modeling. Figure 5c,d show the loadings plot for the PCA model, which illustrates the contribution of each eigenvalue to the model. The larger the coefficient of an eigenvalue, the greater its contribution to the model. Eigenvalues can be decomposed by identifying those with larger coefficients to reduce the dimensionality of the data.

**Figure 6:**
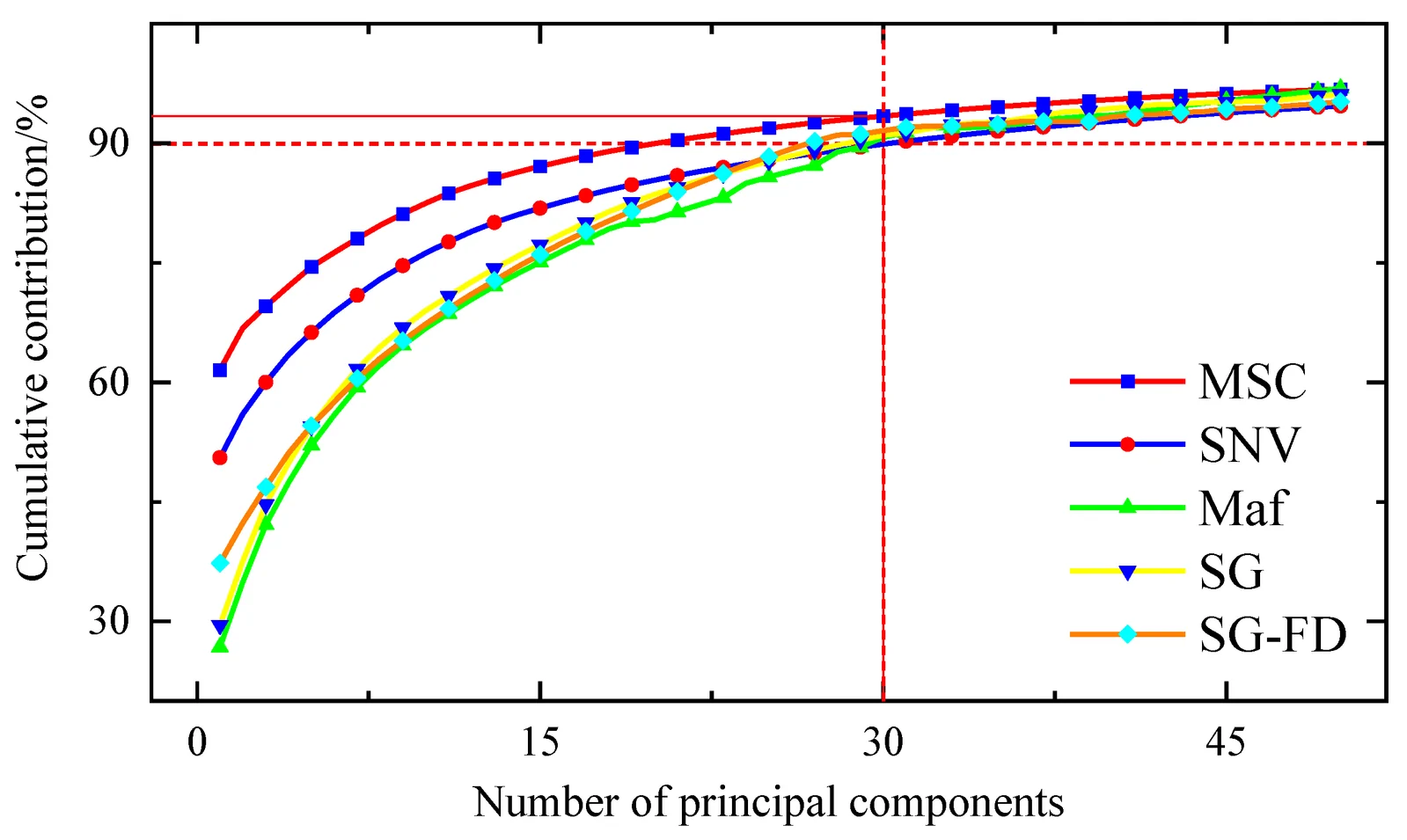
Cumulative contribution rate for the first 50 dimensions.

Since the contribution rates of the first, second, and third principal components are 61.6%, 5.3%, and 2.7%, respectively, their combined contribution accounts for only 69.6% of the vibration information, making it difficult to reliably decompose the eigenvalues. To ensure that the majority of the vibration information is represented, it is necessary to select a higher number of dimensions. Using various preprocessing methods for three-point vibration measurement as examples, we calculated the cumulative contribution rates for the first 50 principal components (Figure 6). The cumulative contribution rate of the first 30 dimensions exceeded 90%.

After performing PCA on the vibration data from the blunt end, sharp end, equator, and three-point measurements, we found that the cumulative contribution rate of the first 30 dimensions was sufficient to meet the requirements (Table 2); therefore, we selected these as the feature components.

We used the feature values extracted via PCA to establish SVM, DAC, LDA, KNN, RF, and NB models for detecting egg freshness and calculated the accuracy on the training and test sets (Table 3). The results show that all egg freshness detection models based on PCA-derived feature extraction performed well, and the accuracy of the three-point vibration measurement models showed higher average accuracy than that of the single-point vibration measurement models. Among them, the SG-FD–PCA–NB and SNV–PCA–DAC models, which are based on three-point vibration measurement, achieved the highest accuracy, reaching 95.833%. The near-perfect training accuracy demonstrated by KNN and RF is not due to their superior generalization ability, but rather because both models are capable of fitting the training data very closely, especially when the number of input feature variables is relatively large.

### 3.2. SPA-Based Feature Extraction and Model Development

**Figure 7:**
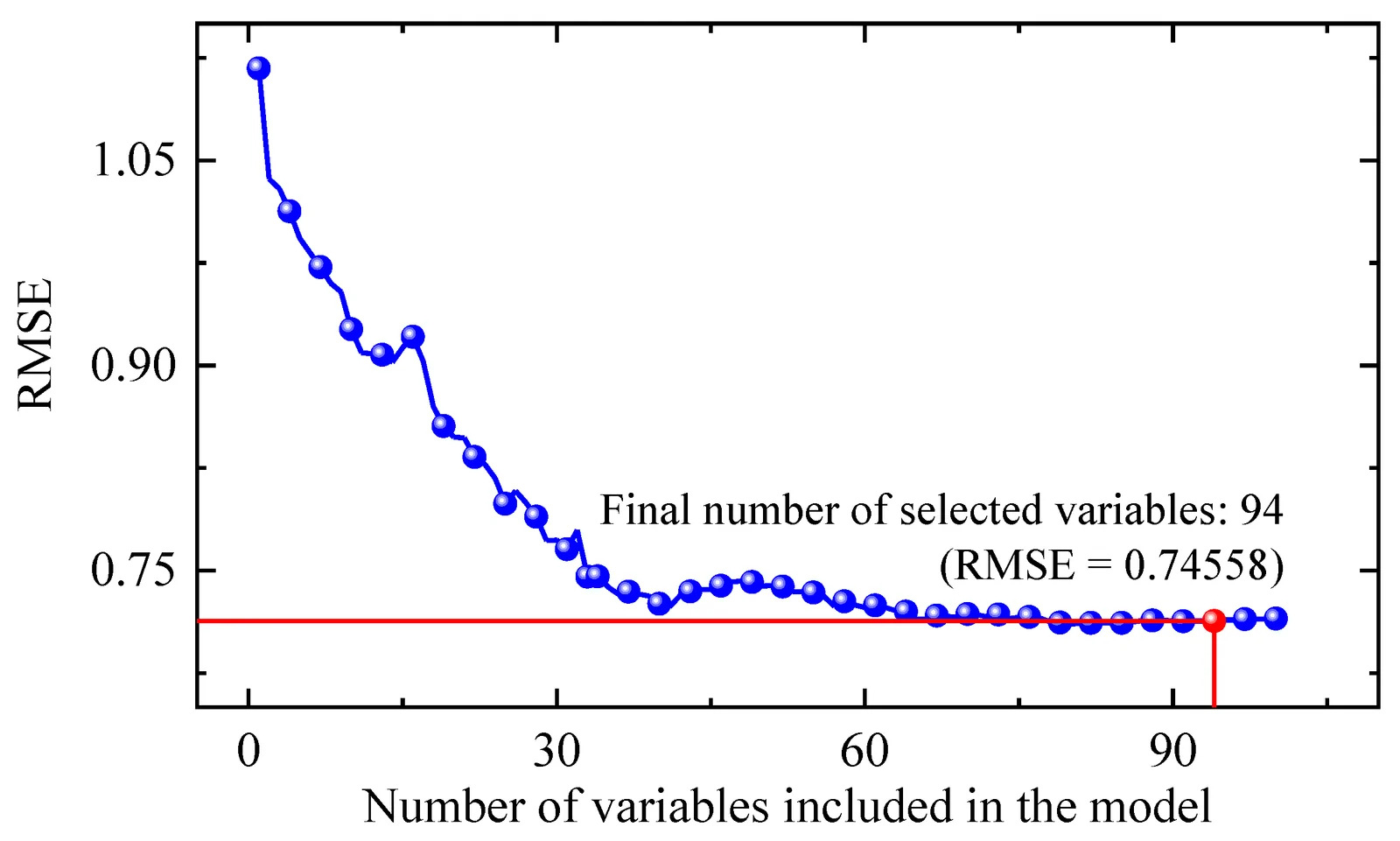
Number of variables in the model (after SG-FD preprocessing).

Because PSD curves contain highly correlated neighboring variables, SPA can identify a compact subset of variables with low multicollinearity while retaining information relevant to egg freshness classification. The SPA eliminates collinearity redundancy, thereby identifying the interval with the least collinearity and the maximum amount of information in the sample [37]. When configuring the SPA, the number of feature values can be set within the range of 5 to 100, with a step size of one. The algorithm performs iterative cycles, selecting the combination of feature values that yields the maximum projected phasor and then using linear regression to calculate the root mean square error (RMSE) for different combinations, until the combination of feature values corresponding to the minimum RMSE is obtained. The three-point vibration test on eggs was conducted, and SPA eigenvalue extraction was performed on the SG-FD preprocessed data (Figure 7). The best results were obtained when the number of feature values was 94, with an RMSE of 0.74558. It should be noted that the relatively large number of selected variables also indicates that the dimensionality of the original power spectral density (PSD) curve has not been completely eliminated.

We used SPA to extract the corresponding number of eigenvectors for different preprocessing methods (Table 4) and establish SVM, DAC, LDA, KNN, RF, and NB models for detecting egg freshness, calculating the accuracy of the training and test sets separately (Table 5). The results show that the SPA-based eigenvalue extraction method performs well, with the DAC detection model demonstrating overall high accuracy, and both MSC and SNV performing well in each model. Furthermore, models based on three-point vibration measurement demonstrated higher accuracy than those based on single-point vibration measurement; among them, the MAF–SPA–DAC model—which utilized three-point vibration measurement—achieved the highest accuracy, reaching 95.833%.

### 3.3. CARS-Based Feature Extraction and Model Development

**Figure 8:**
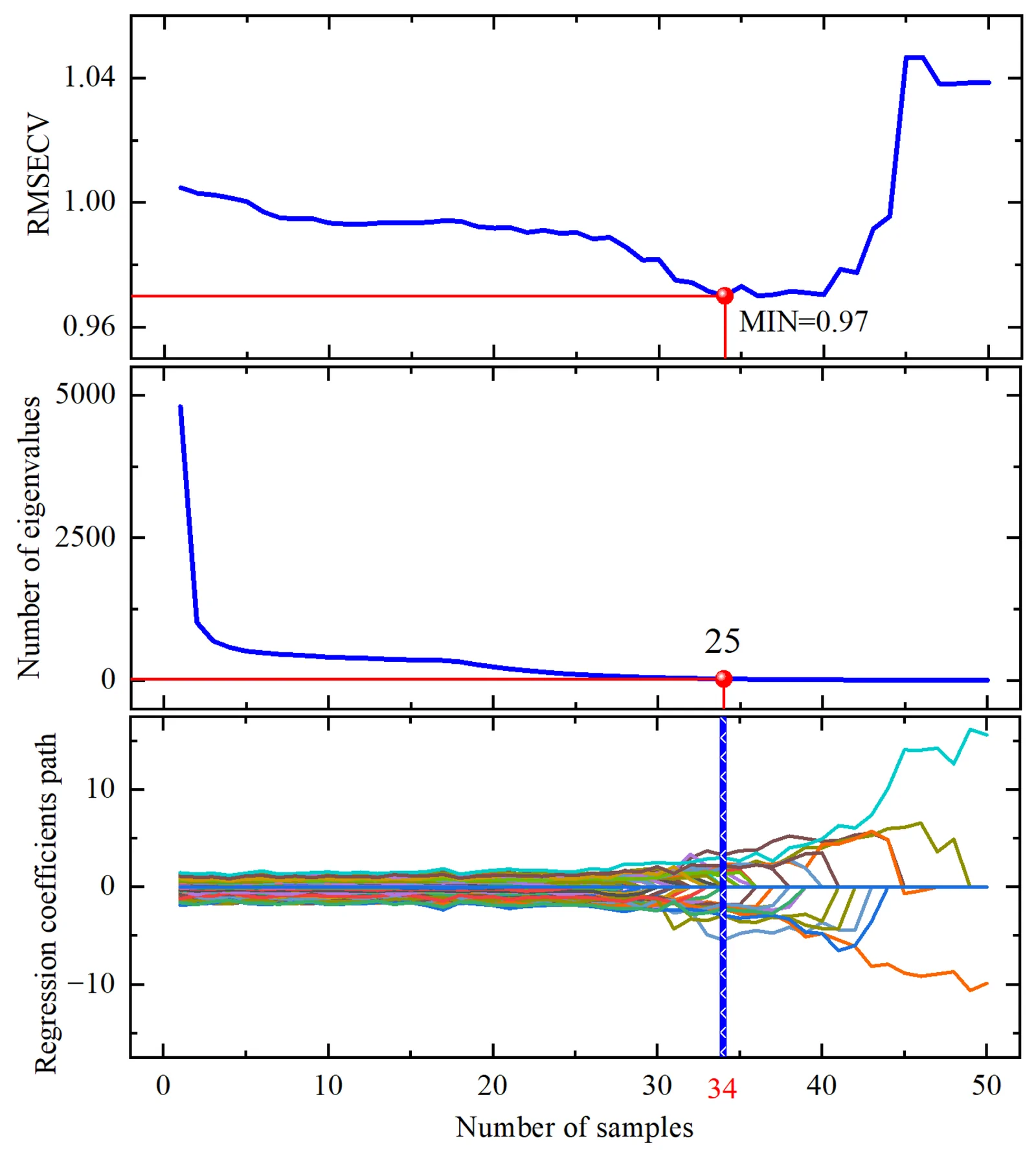
CARS-based feature-value extraction process.

CARS selects spectral values from the bands with larger regression coefficients in the PLS analysis and eliminates spectral values from bands with smaller correlation coefficients [38]. This process selects a set of feature values to represent the entire vibration information, thereby achieving data dimensionality reduction. Random samples were drawn using the Monte Carlo sampling method, with the number of Monte Carlo samples set to 100, and a PLS model was built using five-fold cross-validation. We performed CARS eigenvalue extraction on the three-point vibration measurement data preprocessed using SG-FD (Figure 8). As the number of samples increased, the rate at which the number of retained feature values decreased was initially fast and then slowed down.

During the sampling process from 0 to 100, the root mean square error of cross-validation (RMSECV) decreased slowly, indicating that the removed values had a minor impact on the RMSECV. However, when the number of samples exceeded 34, the RMSECV began to rise, as the feature values had been deleted, resulting in a higher RMSECV. Therefore, the RMSECV was minimized when the number of samples was 34; at this point, the selected feature values were representative of the entire dataset, so the values corresponding to this point were adopted as the feature values. Similarly, the number of feature values after preprocessing using other methods can be determined (Table 6).

After extracting feature values using CARS, we developed SVM, DAC, LDA, KNN, RF, and NB models for egg freshness detection and calculated the accuracy for both the training and test sets (Table 7). The results show that the MAF–CARS–RF model, based on blunt-end vibration measurement, achieved the highest accuracy, reaching 96.667%. Additionally, the MSC–CARS–DAC model based on three-point vibration measurement, the SNV–CARS–SVM model based on equatorial vibration measurement, and the SG-FD–CARS–RF model based on sharp-end vibration measurement performed well, achieving accuracies of 94.167%, 95.000%, and 95.833%, respectively.

### 3.4. Results Analysis

A comparison of the optimal egg freshness detection models under different preprocessing, feature extraction, and modeling methods (Table 8) reveals that laser Doppler vibration measurement technology yields superior results in detecting egg freshness. Among these, the MAF–SPA–DAC model based on three-point vibration measurement and the MAF–CARS–RF model based on blunt-end vibration measurement achieved the highest freshness grading accuracy, reaching 96.7%. Other models, such as the MAF–PCA–DAC, SNV–CARS–SVM, and MSC–SPA–RF based on equatorial vibration measurement, and the SG-FD–CARS–RF based on sharp-end vibration measurement, also achieved high accuracy, at 95% and 95.8%, respectively. Among the evaluated feature-reduction strategies, SPA produced the highest maximum accuracy for the three-point measurement configuration in the present dataset. However, the average performance differences among PCA, SPA, and CARS were relatively small and varied with the measurement location and preprocessing method. Therefore, none of these methods can be considered universally superior.

Furthermore, based on the modeling results presented above (Table 3, Table 5 and Table 7), when comparing the model accuracy corresponding to different vibration measurement methods, it can be observed that, overall, the three-point vibration measurement method yields higher accuracy than the single-point vibration measurement method. Although several processing pipelines achieved similar test-set accuracies, they differ substantially in data acquisition burden, number of retained features, interpretability, and computational complexity. The three-point MAF–SPA–DAC pipeline achieved high accuracy but required measurements at three locations, increasing acquisition time and alignment requirements. In contrast, the blunt-end MAF–CARS–RF pipeline achieved the same accuracy using a single measurement location, which may be more suitable for rapid screening.

## 4. Discussion

Overall, the results demonstrate the feasibility of using LDV for egg-freshness classification under laboratory-scale conditions. The best-performing models achieved an overall accuracy of 96.7% on the evaluated dataset, indicating the potential of LDV as a rapid and nondestructive technique for egg-freshness assessment. However, the experiments were conducted under controlled storage and measurement conditions, which may not fully represent the variability encountered in industrial applications. Factors such as farm of origin, production batch, shell color, egg size, storage environment, and handling conditions may affect the measured vibration responses and classification performance. Therefore, further studies should evaluate the robustness and generalizability of the proposed method using eggs from diverse sources and under more variable storage and measurement conditions.

A further limitation is that only the overall test-set accuracy was retained, whereas sample-level prediction results were unavailable for calculating class-specific performance metrics. Thus, the reported accuracy should be interpreted as an aggregate measure of model performance. Future studies should retain complete sample-level prediction records to enable a more detailed assessment of model performance across different freshness classes.

## 5. Conclusions

This paper investigated a nondestructive method for improving the accuracy of egg-freshness classification based on laser Doppler vibrometry, and analyzed the influence of different measurement locations, signal-preprocessing methods, feature-extraction algorithms, and classifiers on classification performance. The data-processing procedure and conclusions are as follows: (a) Egg-freshness classification models were established by combining different signal-preprocessing methods, feature-extraction algorithms, and machine-learning classifiers. The three-point measurement model based on moving average filtering, successive projections algorithm, and discriminant analysis (MAF–SPA–DAC) achieved the highest classification accuracy of 96.7%. In addition, the blunt-end measurement model based on moving average filtering, competitive adaptive reweighted sampling, and random forest (MAF–CARS–RF) also achieved an accuracy of 96.7%. These results indicate that the vibration response of the eggshell contains characteristic information related to egg freshness. (b) The effects of different measurement configurations on classification performance were further analyzed. The three-point measurement configuration, which included the blunt end, sharp end, and equatorial region, generally provided higher average accuracy because it integrated vibration information from multiple locations. These results demonstrate that the vibration signals collected from eggshells contain characteristic information related to egg freshness. The proposed LDV based method enables rapid and nondestructive classification of eggs with different freshness grades and shows potential for applications in automated egg-quality inspection systems.

## Figures and Tables

**Table 1:** The basic technical parameters of OptoMET Vector.

Index	Value
Frequency Range	DC-500 kHz
Speed resolution	10 nm·s^−1^/√Hz
Maximum speed	2 m/s
Output voltage range	±2 V
Source impedance	50 Ohm
Measuring distance	45 mm–5 m
Laser wavelength	633 nm
Laser power	≤1 mW

**Table 2:** Cumulative contribution rates of the top 30 dimensions.

Vibration Measurement Location	Cumulative Contribution Rate/%			
	MSC	SNV	MAF	SG
Blunt-end	99.0	97.7	96.8	96.3
Sharp-end	98.1	96.6	89.7	89.0
Equatorial	97.8	95.5	90.9	90.2
Three-point	93.4	89.9	90.8	91.0

**Table 3:** The accuracy of the modeling based on PCA.

M.	P.	Training Set Prediction Accuracy/%				Prediction Set Training Accuracy/%			
		B.	S.	E.	T.	B.	S.	E.	T.
SVM	MSC	86.1	76.4	84.6	85.4	66.7	76.7	69.2	81.7
	SNV	100.0	100.0	100.0	100.0	79.2	83.3	70.8	73.3
	MAF	71.4	62.1	66.8	77.5	71.7	69.2	92.5	58.3
	SG	72.9	64.3	73.9	86.9	90.0	87.5	81.7	75.0
	SG-FD	55.0	56.1	58.6	64.3	70.8	69.2	77.5	74.2
DAC	MSC	83.2	77.9	91.4	91.4	90.8	85.8	75.8	88.3
	SNV	92.5	76.8	92.1	76.1	75.0	75.0	85.8	**95.8**
	MAF	75.0	85.7	81.4	89.6	81.7	71.7	**95.0**	94.2
	SG	75.4	71.1	77.5	81.8	**94.7**	64.2	78.3	77.5
	SG-FD	76.1	77.9	77.5	78.6	76.7	85.8	68.3	90.0
LDA	MSC	56.1	68.2	61.8	77.5	70.8	**91.7**	85.8	90.8
	SNV	63.6	65.0	73.9	70.4	87.5	85.0	56.7	90.0
	MAF	67.9	72.1	67.1	68.6	81.7	53.3	90.0	74.2
	SG	63.6	57.1	68.2	74.6	62.5	70.8	80.0	74.2
	SG-FD	70.0	60.0	71.1	78.6	90.8	74.2	82.5	74.2
KNN	MSC	100.0	100.0	100.0	100.0	89.2	72.5	64.2	70.8
	SNV	100.0	100.0	100.0	100.0	69.2	74.2	68.3	78.3
	MAF	100.0	100.0	100.0	100.0	85.8	63.3	82.5	81.7
	SG	100.0	100.0	100.0	100.0	81.7	52.5	63.3	78.3
	SG-FD	100.0	100.0	100.0	100.0	71.7	87.5	78.3	76.7
RF	MSC	100.0	100.0	100.0	99.6	75.8	77.5	94.2	64.2
	SNV	100.0	100.0	100.0	100.0	71.7	75.0	81.7	73.3
	MAF	100.0	100.0	100.0	100.0	80.8	80.8	69.2	93.3
	SG	100.0	99.3	100.0	100.0	85.0	68.3	61.7	75.0
	SG-FD	100.0	100.0	100.0	100.0	87.5	81.7	69.2	77.5
NB	MSC	79.3	74.3	80.0	70.4	73.3	58.3	86.7	90.0
	SNV	66.1	70.0	83.6	85.7	75.8	59.2	74.2	89.2
	MAF	76.1	66.4	61.0	62.9	76.7	74.2	65.0	77.5
	SG	63.6	76.4	77.1	69.6	65.0	**91.7**	60.8	59.2
	SG-FD	65.4	66.2	72.1	66.4	73.3	64.2	71.7	**95.8**
MAX		100.0	100.0	100.0	100.0	94.7	91.7	95.0	95.8
AVERAGE		82.6	81.4	84.5	85.7	79.0	74.7	76.6	**80.3**

M., model; P., preprocessing methods; B., blunt-end vibration measurement; S., sharp-end vibration measurement; E., equatorial vibration measurement; T., three-point vibration measurement; SVM, support vector machine; DAC, discriminant analysis classifier; LDA, linear discriminant analysis; KNN, k-nearest neighbor; RF, random forest; NB, naive Bayes; MSC, multiplicative scatter correction; SNV, standard normal variate; MAF, moving average filtering; SG, Savitzky–Golay smoothing; SG-FD, first-order Savitzky–Golay differentiation; bold values indicate the highest prediction accuracy within the corresponding vibration-measurement condition.

**Table 4:** Number of feature values after SPA processing.

Vibration Measurement Location	Number of Feature Values			
	MSC	SNV	MAF	SG
Blunt-end	46	59	43	68
Sharp-end	43	43	34	48
Equatorial	49	65	48	35
Three-point	63	96	80	65

**Table 5:** The accuracy of the modeling based on SPA.

M.	P.	Training Set Prediction Accuracy/%				Prediction Set Training Accuracy/%			
		B.	S.	E.	T.	B.	S.	E.	T.
SVM	MSC	83.6	76.4	81.1	85.0	64.2	75.0	70.8	85.8
	SNV	99.6	97.5	99.6	100.0	80.0	82.5	69.2	75.8
	MAF	69.6	61.1	63.2	76.8	68.3	71.7	90.8	60.8
	SG	76.1	62.5	77.5	83.2	86.7	88.3	85.8	73.3
	SG-FD	52.1	56.1	56.8	65.4	70.0	67.5	74.2	76.7
DAC	MSC	82.9	80.0	92.1	93.9	92.5	85.8	74.2	92.5
	SNV	91.8	75.0	88.9	75.7	77.5	75.8	86.7	95.0
	MAF	95.4	86.8	84.3	92.1	79.2	73.3	94.2	**96.7**
	SG	97.9	72.9	78.2	81.1	**95.0**	68.3	75.0	75.0
	SG-FD	93.8	77.5	77.1	80.0	75.8	85.8	65.0	86.7
LDA	MSC	59.3	71.1	63.9	80.7	73.3	87.5	83.3	90.0
	SNV	62.1	65.0	75.7	71.8	86.7	86.7	59.2	86.7
	MAF	68.2	71.1	67.5	67.9	80.8	55.8	91.7	77.5
	SG	64.3	55.7	68.9	72.1	62.5	66.7	79.2	74.2
	SG-FD	72.9	62.5	71.4	82.1	89.2	73.3	82.5	74.2
KNN	MSC	96.4	96.8	98.2	100.0	90.8	75.8	65.8	70.8
	SNV	97.1	96.4	96.8	98.9	65.8	74.2	70.0	75.8
	MAF	97.1	100.0	96.4	100.0	82.5	63.3	80.8	78.3
	SG	100.0	98.2	97.1	100.0	77.5	50.0	65.0	79.2
	SG-FD	96.8	97.9	100.0	100.0	75.8	87.5	80.8	72.5
RF	MSC	98.6	97.9	100.0	100.0	80.0	80.8	**95.0**	68.3
	SNV	99.6	100.0	99.3	100.0	70.0	73.3	81.7	71.7
	MAF	100.0	100.0	100.0	100.0	82.5	79.2	66.7	95.8
	SG	100.0	98.6	98.6	98.9	83.3	70.0	62.5	75.0
	SG-FD	99.6	96.8	100.0	100.0	88.3	80.0	73.3	76.7
NB	MSC	77.9	76.8	78.6	67.5	69.167	58.3	85.0	86.7
	SNV	66.4	67.5	81.8	87.9	80.0	56.7	74.2	87.5
	MAF	76.1	69.3	60.0	63.9	74.2	75.8	60.8	73.3
	SG	63.6	73.2	78.2	67.1	66.7	**95.0**	58.3	59.2
	SG-FD	67.5	66.8	72.9	68.2	75.0	64.2	75.8	95.0
MAX		100.0	100.0	100.0	100.0	95.0	95.0	95.0	96.7
AVERAGE		82.1	80.9	84.0	85.8	78.5	74.9	76.5	**80.1**

M., model; P., preprocessing methods; B., blunt-end vibration measurement; S., sharp-end vibration measurement; E., equatorial vibration measurement; T., three-point vibration measurement; SVM, support vector machine; DAC, discriminant analysis classifier; LDA, linear discriminant analysis; KNN, k-nearest neighbor; RF, random forest; NB, naive Bayes; MSC, multiplicative scatter correction; SNV, standard normal variate; MAF, moving average filtering; SG, Savitzky–Golay smoothing; SG-FD, first-order Savitzky–Golay differentiation; bold values indicate the highest prediction accuracy within the corresponding vibration-measurement condition.

**Table 6:** Number of feature values after CARS processing.

Vibration Measurement Location	Number of Feature Values			
	MSC	SNV	MAF	SG
Blunt-end	61	53	213	231
Sharp-end	6	40	120	10
Equatorial	7	16	18	69
Three-point	22	56	8	10

**Table 7:** The accuracy of the modeling based on CARS.

M.	P.	Training Set Prediction Accuracy/%				Prediction Set Training Accuracy/%			
		B.	S.	E.	T.	B.	S.	E.	T.
SVM	MSC	76.1	67.9	71.4	73.9	65.0	70.8	60.0	78.3
	SNV	100.0	100.0	100.0	100.0	94.2	93.3	**95.0**	81.7
	MAF	70.7	70.0	70.7	75.0	74.2	55.0	80.8	75.0
	SG	83.2	66.8	76.8	57.6	86.7	58.3	84.2	66.7
	SG-FD	55.7	58.2	57.9	58.2	60.8	57.5	80.8	75.0
DAC	MSC	100.0	61.4	82.5	77.5	88.3	69.2	86.7	**94.2**
	SNV	100.0	100.0	90.7	94.3	95.0	75.8	85.8	90.0
	MAF	100.0	100.0	83.9	78.6	77.5	86.7	79.2	62.5
	SG	100.0	64.3	100.0	73.6	93.3	65.8	90.8	70.0
	SG-FD	100.0	80.7	100.0	72.9	85.8	80.8	90.0	93.3
LDA	MSC	64.6	42.5	52.9	70.4	68.3	51.7	70.8	82.5
	SNV	66.1	66.4	68.6	70.4	74.2	55.8	70.0	80.8
	MAF	100.0	100.0	59.6	40.7	43.3	52.5	60.0	74.2
	SG	100.0	60.0	55.7	51.1	67.5	68.3	75.8	72.5
	SG-FD	80.0	55.4	53.9	56.4	55.8	57.5	69.2	89.2
KNN	MSC	100.0	100.0	100.0	100.0	65.0	75.8	69.2	81.7
	SNV	100.0	100.0	100.0	100.0	84.2	75.8	94.2	66.7
	MAF	100.0	100.0	100.0	100.0	70.0	84.2	75.0	73.3
	SG	100.0	100.0	100.0	100.0	83.3	70.8	74.2	82.5
	SG-FD	100.0	100.0	100.0	100.0	85.8	63.3	95.0	86.7
RF	MSC	99.6	99.3	99.6	100.0	75.8	68.3	75.8	70.8
	SNV	100.0	100.0	100.0	100.0	91.7	92.5	83.3	87.5
	MAF	100.0	100.0	100.0	100.0	**96.7**	90.0	59.2	64.2
	SG	100.0	99.6	100.0	100.0	94.2	73.3	64.2	63.3
	SG-FD	100.0	100.0	100.0	99.6	94.2	**95.8**	90.0	86.7
NB	MSC	63.6	63.2	76.9	62.5	80.8	69.2	65.0	60.8
	SNV	55.7	70.4	76.9	75.7	59.2	66.7	80.0	88.3
	MAF	63.6	63.9	71.4	70.0	67.5	59.2	64.2	78.3
	SG	63.9	56.1	76.1	65.4	50.8	77.5	54.2	61.7
	SG-FD	68.6	83.9	76.4	71.8	76.7	69.2	85.8	88.3
MAX		100.0	100.0	100.0	100.0	**96.7**	95.8	95.0	94.2
AVERAGE		87.5	81.6	83.9	80.5	77.5	71.8	77.5	**78.1**

M., model; P., preprocessing methods; B., blunt-end vibration measurement; S., sharp-end vibration measurement; E., equatorial vibration measurement; T., three-point vibration measurement; SVM, support vector machine; DAC, discriminant analysis classifier; LDA, linear discriminant analysis; KNN, k-nearest neighbor; RF, random forest; NB, naive Bayes; MSC, multiplicative scatter correction; SNV, standard normal variate; MAF, moving average filtering; SG, Savitzky–Golay smoothing; SG-FD, first-order Savitzky–Golay differentiation; bold values indicate the highest prediction accuracy within the corresponding vibration-measurement condition.

**Table 8:** The optimal model and accuracy for freshness detection.

Vibration Measurement Location	Feature Extraction Methods	Average Model Accuracy/%	Optimal Model	Optimal Accuracy/%
Blunt-end	PCA	79.0	MAF–CARS–RF	96.7
	SPA	78.5		
	CARS	77.5		
Sharp-end	PCA	74.7	SG-FD–CARS–RF	95.8
	SPA	74.9		
	CARS	71.8		
Equatorial	PCA	76.6	MAF–PCA–DAC	95.0
	SPA	76.5	MSC–SPA–RF	
	CARS	77.5	SNV–CARS–SVM	
Three-point	PCA	80.3	MAF–SPA–DAC	96.7
	SPA	80.1		
	CARS	78.1		

## Data Availability

The data presented in this study are available from the corresponding author upon reasonable request.

## Abbreviations

The following abbreviations are used in this manuscript:

LDV	Laser Doppler vibrometry
*HU*	Haugh unit
BEVM	Blunt-end vibration measurement
SEVM	Sharp-end vibration measurement
EVM	Equator vibration measurement
PSD	Power spectrum density
FFT	Fast Fourier transform
MSC	Multiplicative scatter correction
SNV	Standardized normal variate
MAF	Moving average filter
SG	Savitzky–Golay smoothing
SG-FD	Savitzky–Golay first derivative
PCA	Principal components analysis
SPA	Successive projections algorithm
CARS	Competitive adaptive reweighted sampling
SVM	Support vector machines
DAC	Discriminant analysis classifier
LDA	Latent Dirichlet allocation
KNN	K-nearest neighbor
RF	Random forest
NB	Naive Bayes
RMSE	Root mean square error
RMSECV	Root mean square error of cross-validation

## References

[1] Renzone G., Novi G., Scaloni A., Arena S. (2021). Monitoring aging of hen egg by integrated quantitative peptidomic procedures. Food Res. Int..

[2] Scott T.A., Silversides F.G. (2000). The effect of storage and strain of hen on egg quality. Poult. Sci..

[3] Silversides F.G., Scott T.A. (2001). Effect of storage and layer age on quality of eggs from two lines of hens. Poult. Sci..

[4] Feddern V., Prá M.C.D., Mores R., Nicoloso R.D.S., Coldebella A., Abreu P.G.D. (2017). Egg quality assessment at different storage conditions, seasons and laying hen strains. Ciênc. Agrotecnol..

[5] Karoui R., Kemps B., Bamelis F., De Ketelaere B., Decuypere E., De Baerdemaeker J. (2006). Methods to evaluate egg freshness in research and industry: A review. Eur. Food Res. Technol..

[6] Liu Y., Ying Y., Ouyang A., Li Y. (2007). Measurement of internal quality in chicken eggs using visible transmittance spectroscopy technology. Food Control.

[7] Zita L., Jeníková M., Härtlová H. (2018). Effect of housing system on egg quality and the concentration of cholesterol in egg yolk and blood of hens of native resources of the Czech Republic and Slovakia. J. Appl. Poult. Res..

[8] Narushin V.G., Romanov M.N., Griffin D.K. (2021). A novel Egg Quality Index as an alternative to Haugh unit score. J. Food Eng..

[9] Yang N., Jin Y., Wang H., Duan X., Xu B., Jin Z., Xu X. (2015). Evaluation of conductivity and moisture content of eggs during storage by using transformer method. J. Food Eng..

[10] Nematinia E., Abdanan Mehdizadeh S. (2018). Assessment of egg freshness by prediction of Haugh unit and albumen pH using an artificial neural network. J. Food Meas. Charact..

[11] Harnsoongnoen S., Jaroensuk N. (2021). The grades and freshness assessment of eggs based on density detection using machine vision and weighing sensor. Sci. Rep..

[12] Bambilla R.L.M., Lee M.J.B., Valiente F.L. (2025). Freshness Condition Detection, Size Classification with Weight Prediction and Sortation of Philippine Egg Produce Using YOLOv8 and OpenCV. 2025 17th International Conference on Computer and Automation Engineering (ICCAE).

[13] Dutta R., Hines E.L., Gardner J.W., Udrea D.D., Boilot P. (2003). Non-destructive egg freshness determination: An electronic nose based approach. Meas. Sci. Technol..

[14] Yongwei W., Wang J., Zhou B., Lu Q. (2009). Monitoring storage time and quality attribute of egg based on electronic nose. Anal. Chim. Acta.

[15] Yimenu S.M., Kim J.Y., Kim B.S. (2017). Prediction of egg freshness during storage using electronic nose. Poult. Sci..

[16] Dai D., Jiang T., Lu W., Shen X., Xiu R., Zhang J. (2020). Nondestructive Detection for Egg Freshness Based on Hyperspectral Scattering Image Combined with Ensemble Learning. Sensors.

[17] Cruz-Tirado J.P., Lucimar Da Silva Medeiros M., Barbin D.F. (2021). On-line monitoring of egg freshness using a portable NIR spectrometer in tandem with machine learning. J. Food Eng..

[18] Davari M., Bahreini M., Sabzevari Z. (2024). Developing a non-destructive method for the detection of egg quality and freshness using micro-Raman spectroscopy. Appl. Food Res..

[19] Ragni L., Cevoli C., Berardinelli A. (2010). A waveguide technique for non-destructive determination of egg quality parameters. J. Food Eng..

[20] Akbarzadeh N., Mireei S.A., Askari G., Mahdavi A.H. (2019). Microwave spectroscopy based on the waveguide technique for the nondestructive freshness evaluation of egg. Food Chem..

[21] Soltani M., Omid M. (2015). Detection of poultry egg freshness by dielectric spectroscopy and machine learning techniques. LWT—Food Sci. Technol..

[22] Hu M., Zhao M., Qi L., Li D., Wang X., Li Z., Zhao S., Fan K. (2024). Non-destructive inspection method for egg freshness evaluation via low-field nuclear magnetic resonance technology. J. Food Meas. Charact..

[23] Quattrocchi A., Freni F., Montanini R., Turrisi S., Zappa E. (2022). Development, Validation and Preliminary Experiments of a Measuring Technique for Eggs Aging Estimation Based on Pulse Phase Thermography. Sensors.

[24] Nakaguchi V.M., Ahamed T. (2022). Fast and Non-Destructive Quail Egg Freshness Assessment Using a Thermal Camera and Deep Learning-Based Air Cell Detection Algorithms for the Revalidation of the Expiration Date of Eggs. Sensors.

[25] Nakaguchi V.M., Liu Z., Abeyrathna R.M.R.D., Kuo Y., Genkawa T., Ahamed T. (2026). A thermal machine vision sensing system for quality assurance of quail eggs—Freshness assessment and grading. Comput. Electron. Agric..

[26] Muramatsu N., Sakurai N., Wada N., Yamamoto R., Takahara T., Ogata T., Tanaka K., Asakura T., Ishikawa-Takano Y., Nevins D.J. (1999). Evaluation of fruit tissue texture and internal disorders by laser Doppler detection. Postharvest Biol. Technol..

[27] Terasaki S., Sakurai N., Kuroki S., Yamamoto R., Nevins D.J. (2013). A new descriptive method for fruit firmness changes with various softening patterns of kiwifruit. Postharvest Biol. Technol..

[28] Landahl S., Terry L.A. (2022). Detection of internal defects in onion bulbs by means of single-point and scanning laser Doppler vibrometry. Biosyst. Eng..

[29] Loffredi E., Grassi S., Alamprese C. (2021). Spectroscopic approaches for non-destructive shell egg quality and freshness evaluation: Opportunities and challenges. Food Control.

[30] Haugh R. (1937). The Haugh Unit for Measuring Egg Quality. US Egg Poult. Mag..

[31] Narushin V.G. (1997). Non-destructive measurements of egg parameters and quality characteristics. World’s Poult. Sci. J..

[32] Adrian R.J., Yao C.S. (1986). Power spectra of fluid velocities measured by laser Doppler velocimetry. Exp. Fluids.

[33] Zhao J., Fang Y., Chu G., Yan H., Hu L., Huang L. (2020). Identification of Leaf-Scale Wheat Powdery Mildew (*Blumeria graminis* f. sp. Tritici) Combining Hyperspectral Imaging and an SVM Classifier. Plants.

[34] Mishra P., Lohumi S., Ahmad Khan H., Nordon A. (2020). Close-range hyperspectral imaging of whole plants for digital phenotyping: Recent applications and illumination correction approaches. Comput. Electron. Agric..

[35] Javid A., Salahshoor K. (2025). Real-time leak detection system: A hybrid model of moving average and Kalman filters with long short-term memory (LSTM) on the Internet of things (IoT) platform. Process Saf. Environ..

[36] Zheng K., Zhang X., Tong P., Yao Y., Du Y. (2015). Pretreating near infrared spectra with fractional order Savitzky–Golay differentiation (FOSGD). Chin. Chem. Lett..

[37] Wang Z., Ding J., Zhang Z. (2022). Estimation of Soil Organic Matter in Arid Zones with Coupled Environmental Variables and Spectral Features. Sensors.

[38] Deneva V., Bakardzhiyski I., Bambalov K., Antonova D., Tsobanova D., Bambalov V., Cozzolino D., Antonov L. (2019). Using Raman Spectroscopy as a Fast Tool to Classify and Analyze Bulgarian Wines—A Feasibility Study. Molecules.

[39] Goudarzi N., Goodarzi M., Araujo M.C.U., Galvo R.K.H. (2009). QSPR Modeling of Soil Sorption Coefficients (KOC) of Pesticides Using SPA-ANN and SPA-MLR. J. Agric. Food Chem..

[40] Han Z., Li B., Wang Q., Yang A., Liu Y., Camara J.S. (2022). Detection Storage Time of Mild Bruise’s Loquats Using Hyperspectral Imaging. J. Spectrosc..

[41] Leong Y.X., Lee Y.H., Koh C.S.L., Phan-Quang G.C., Han X., Phang I.Y., Ling X.Y. (2021). Surface-Enhanced Raman Scattering (SERS) Taster: A Machine-Learning-Driven Multireceptor Platform for Multiplex Profiling of Wine Flavors. Nano Lett..

[42] Sullivan S.Z., Schmitt P.D., Muir R.D., DeWalt E.L., Simpson G.J. (2014). Digital deconvolution filter derived from linear discriminant analysis and application for multiphoton fluorescence microscopy. Anal. Chem..

[43] Xin Z., Jun S., Xiaohong W., Bing L., Ning Y., Chunxia D. (2019). Research on moldy tea feature classification based on WKNN algorithm and NIR hyperspectral imaging. Spectrochim. Acta Part A Mol. Biomol. Spectrosc..

[44] Sai K., Sood N., Saini I. (2022). Abiotic stress classification through spectral analysis of enhanced electrophysiological signals of plants. Biosyst. Eng..

[45] Ghamisi P., Plaza J., Chen Y., Li J., Plaza A. (2017). Advanced Spectral Classifiers for Hyperspectral Images: A review. IEEE Geosci. Remote Sens. Mag..

[46] Susič N., Žibrat U., Širca S., Strajnar P., Razinger J., Knapič M., Vončina A., Urek G., Gerič Stare B. (2018). Discrimination between abiotic and biotic drought stress in tomatoes using hyperspectral imaging. Sens. Actuators B Chem..

